# Exercise Training Alleviates Cardiac Fibrosis through Increasing Fibroblast Growth Factor 21 and Regulating TGF-β1-Smad2/3-MMP2/9 Signaling in Mice with Myocardial Infarction

**DOI:** 10.3390/ijms222212341

**Published:** 2021-11-15

**Authors:** Yixuan Ma, Yixin Kuang, Wenyan Bo, Qiaoqin Liang, Wenfei Zhu, Mengxin Cai, Zhenjun Tian

**Affiliations:** 1 Institute of Sports Biology, College of Physical Education, Shaanxi Normal University, Xi’an 710119, China; mayixuan07@snnu.edu.cn (Y.M.); kyx479336797@163.com (Y.K.); boweny9909@snnu.edu.cn (W.B.); liangqq@snnu.edu.cn (Q.L.); wzhu@snnu.edu.cn (W.Z.); 2 College of Physical Education and Health, Guizhou Minzu University, Guiyang 550025, China

**Keywords:** exercise training, myocardial infarction, cardiac fibrosis, fibroblast growth factor 21

## Abstract

Exercise training has been reported to alleviate cardiac fibrosis and ameliorate heart dysfunction after myocardial infarction (MI), but the molecular mechanism is still not fully clarified. Fibroblast growth factor 21 (FGF21) exerts a protective effect on the infarcted heart. This study investigates whether exercise training could increase FGF21 protein expression and regulate the transforming growth factor-β1 (TGF-β1)-Smad2/3-MMP2/9 signaling pathway to alleviate cardiac fibrosis following MI. Male wild type (WT) C57BL/6J mice and *Fgf21* knockout (*Fgf21* KO) mice were used to establish the MI model and subjected to five weeks of different types of exercise training. Both aerobic exercise training (AET) and resistance exercise training (RET) significantly alleviated cardiac dysfunction and fibrosis, up-regulated FGF21 protein expression, inhibited the activation of TGF-β1-Smad2/3-MMP2/9 signaling pathway and collagen production, and meanwhile, enhanced antioxidant capacity and reduced cell apoptosis in the infarcted heart. In contrast, knockout of *Fgf21* weakened the cardioprotective effects of AET after MI. In vitro, cardiac fibroblasts (CFs) were isolated from neonatal mice hearts and treated with H_2_O_2_ (100 μM, 6 h). Recombinant human FGF21 (rhFGF21, 100 ng/mL, 15 h) and/or 5-Aminoimidazole-4-carboxamide ribonucleotide (AICAR, 1 mM, 15 h) inhibited H_2_O_2_-induced activation of the TGF-β1-Smad2/3-MMP2/9 signaling pathway, promoted CFs apoptosis and reduced collagen production. In conclusion, exercise training increases FGF21 protein expression, inactivates the TGF-β1-Smad2/3-MMP2/9 signaling pathway, alleviates cardiac fibrosis, oxidative stress, and cell apoptosis, and finally improves cardiac function in mice with MI. FGF21 plays an important role in the anti-fibrosis effect of exercise training.

## 1. Introduction

Myocardial infarction (MI) is one of the leading causes of death worldwide [1,2]. Myocardial ischemia and hypoxia after MI induce a sharp production of reactive oxygen species (ROS), leading to oxidative stress injury and numbers of cardiomyocytes necrosis and apoptosis. In contrast, with the activation of cardiac fibroblasts (CFs) and increased collagen deposition, alternative fibrosis and scar tissue occur in the infarcted area, resulting in cardiac pathological remodeling and dysfunction [3,4,5]. Ameliorating cardiac fibrosis and inhibiting cardiomyocytes apoptosis would be key therapeutic targets to improve cardiac function following MI. Increasing data from clinic and animal research suggests that exercise training is an effective strategy in improving cardiac function [6,7,8,9,10,11,12]. Exercise training has beneficial effects on preventing or reversing pathological cardiac remodeling by reducing collagen production [13]. However, the possible mechanisms of exercise training on alleviating cardiac fibrosis after MI need further study.

Cardiac fibrosis is a pathological remodeling process of the extracellular matrix (ECM). Transforming growth factor-β (TGF-β) is probably the best-characterized pro-fibrotic growth factor [4,14]. Among the three TGF-β isoforms (1, 2, and 3), TGF-β1 could be activated by ROS, and plays an important role in the injured heart [15,16]. It has been reported that activated TGF-β1 binds to its receptors and induced collagen production through the Smad signaling cascades [15,17]. In addition, the production of matrix metalloproteinases (MMPs) by CFs degrades the ECM and allows cell migration into the injured area. Consequently, the activation of TGF-β-Smad2/3-MMPs signal pathway eventually leads to the formation of scar tissue. One study showed that exercise training could inactivate TGF-β1/Smad signaling pathway to alleviate cardiac fibrosis in MI rats [13]. However, the targets of exercise training in regulating TGF-β1/Smad signaling pathway and improving pathological remodeling in the infarcted heart are not clear.

**Figure 1:**
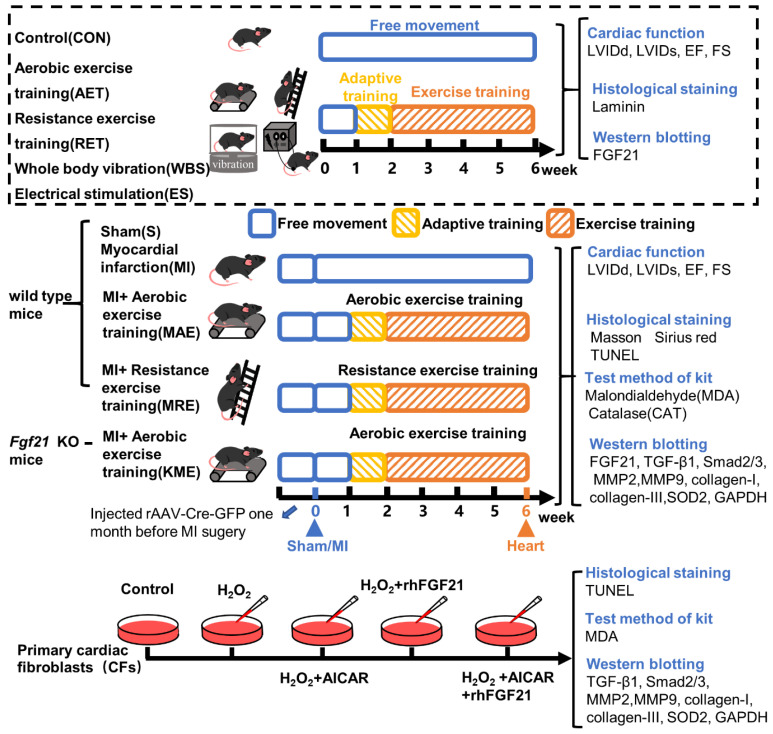
Schematic diagram of experimental design.

Fibroblast growth factor 21 (FGF21) is an endocrine factor, expressed in liver, adipose tissue, skeletal muscle, kidney, heart, blood vessels, and so on [18,19]. It has been demonstrated that FGF21 has a wide range of biological functions, including regulation of glucose and lipid homeostasis [18,20]. In multiple cardiovascular diseases, FGF21 has been reported to enhance the antioxidant capacity, regulate mitochondrial function, inhibit cell apoptosis and pathological cardiac hypertrophy, as well as reduce cardiac fibrosis [21,22,23,24,25]. FGF21 could down-regulate TGF-β1 and induce nuclear translocation of Smad2/3 to reduce collagen precipitation and renal fibrosis [26]. Whether FGF21 could inhibit MI-induced cardiac fibrosis by regulating TGF-β1/Smad/MMPs signaling pathway is worth confirming. A variety of exercise modes could up-regulate the FGF21 level [27,28,29]. In this study we detected whether FGF21 could function as a target of exercise training during the exercise-induced cardioprotective effect, especially in the alleviation of cardiac fibrosis. We performed in vivo and in vitro experiments using wild type (WT) C57BL/6J mice, *Fgf21* knockout mice (*Fgf21* KO) and CFs to explore the role and possible mechanism of FGF21 in the anti-fibrosis effect of exercise training in mice with MI. Moreover, we compared the effects of different types of exercise training on FGF21 protein expression, cardiac function and histological changes in normal and infarcted mice. The experimental design was shown in Figure 1.

## 2. Results

### 2.1. Exercise Training Up-Regulated FGF21 Expression and Alleviated Cardiac Dysfunction and Cardiac Fibrosis in the Infarcted Heart

At first, 10-month-old mice were subjected to four types of exercise training separately, including aerobic exercise training (AET), resistance exercise training (RET), whole body vibration exercise group (WBV), and skeletal muscle electrical stimulation group (ES). Compared with control mice, AET and RET increased the cross-sectional area (CSA) of cardiomyocytes (both *p* < 0.01), heart weight (both *p* < 0.01), and the ratio of heart weight/body weight (HW/BW, both *p* < 0.01) of mice in AET and RET groups; AET, RET, WBV and ES all increased the ratio of heart weight/tibial length (HW/TL, *p* < 0.01 for AET and RET, *p* < 0.05 for WBV and ES), left ventricular ejection fraction (EF) and left ventricular fractional shortening (FS, *p* < 0.01 for AET and RET, *p* < 0.05 for WBV and ES), reduced left ventricular internal diameter at end diastole (LVIDd) and left ventricular internal diameter at end systole (LVIDs) (*p* < 0.01 for AET and RET, *p* < 0.05 for WBV and ES) (Figure S1). According to these results, we chose AET and RET to intervene the MI animals.

**Figure 2:**
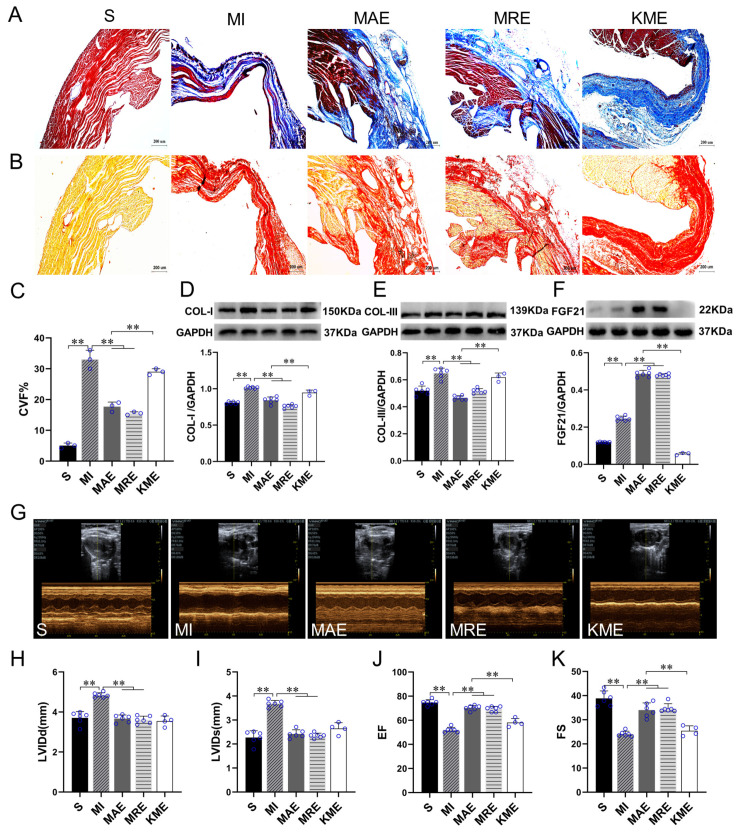
Knockout of *Fgf21* attenuated exercise-induced inhibition of cardiac fibrosis and improvement of heart function following MI. (**A**) Masson staining, cardiomyocytes (red), collagen fibers (blue) and nuclei (brown) are shown, scale bar = 200 µm; (**B**) Sirius red staining, cardiomyocytes (yellow), collagen fibers (red) and nuclei (brown) are shown, scale bar = 200 µm; (**C**) Statistical analysis of CVF, the level of cardiac fibrosis in the infarcted heart was evaluated by the Image Pro Plus analysis software. CVF was calculated as collagen area/total area of myocardial tissue × 100%; (**D**–**F**) Protein expressions of COL-I, COL-III and FGF21 in the myocardium; (**G**–**K**) Statistical analysis of echocardiographic results and cardiac function in mice. Data are expressed as mean ± Standard Deviation (SD), *n* = 8 in S, MI, MAE and MRE groups and *n* = 4 in KME group. Statistical method: one-way analysis of variance (ANOVA). ** *p* < 0.01. CVF, collagen volume fraction; COL- I, collagen type I; COL-III, collagen type III; LVIDd, left ventricular internal diameter at end diastole; LVIDs, left ventricular internal diameter at end systole; EF, left ventricular ejection fraction; FS, left ventricular fractional shortening; S, Sham group; MI, MI-sedentary group; MAE, post-MI aerobic exercise training group; MRE, post-MI resistance exercise training group; and KME, the *Fgf21* KO mice with aerobic exercise training group.

We detected the cardiac function and fibrosis to verify the effects of exercise training in the mice with MI. Masson staining, sirius red staining and collagen content were detected to assess the degree of cardiac fibrosis. Major components of the myocardial ECM are collagen type I (COL-I) and type III (COL-III) [30]. Therefore, we detected the protein expressions of COL-I and COL-III. The results showed that compared with the Sham-operated (S) group, replacement fibrosis occurred in the infarcted heart of MI mice, accompanied with increased collagen volume fraction (CVF) level (*p* < 0.01) and the protein expressions of COL-I (*p* < 0.01) and COL-III (*p* < 0.01), meanwhile, LVIDd (*p* < 0.01) and LVIDs (*p* < 0.01) significantly increased, and EF (*p* < 0.01) and FS (*p* < 0.01) decreased in the MI-sedentary (MI) groups. Compared with the MI group, AET and RET reduced the CVF level (both *p* < 0.01) and the protein expressions of COL-I (both *p* < 0.01) and COL-III (both *p* < 0.01) as well as the levels of LVIDd (both *p* < 0.01) and LVIDs (both *p* < 0.01), increased EF (both *p* < 0.01) and FS (both *p* < 0.01) of the infarcted hearts in the MAE and MRE groups (Figure 2A–E,G–K). These results indicated that AET and RET inhibited cardiac fibrosis and improved cardiac function in mice with MI.

FGF21 exerts a protective effect in multiple disease models [22,23,24]. In this study, we detected the protein expression of FGF21 in the heart. In the normal mice, AET (*p* < 0.01), RET (*p* < 0.01), WBV (*p* < 0.05) and ES (*p* < 0.01) increased FGF21 protein expression significantly (Figure S2A). Moreover, Pearson correlation analysis showed significant correlations between FGF21 level and LVIDd (r = −0.553, *p* < 0.01), LVIDs (r = −0.734, *p* < 0.01), EF (r = 0.829, *p* < 0.01) and FS (r = 0.799, *p* < 0.01) (Figure S2B–E). In the injured heart, compared with the S group, the FGF21 protein expression increased (*p* < 0.01) significantly in the MI group. Compared with the MI group, AET and RET further up-regulated the FGF21 protein expression (both *p* < 0.01) in the infarcted heart (Figure 2F). Based on these results, we speculated that FGF21 would play an important role in the cardioprotective effects of exercise.

Then, we used *Fgf21* KO mice to establish the MI model and evaluate the role of FGF21 in the cardioprotective effects of exercise. Recombinant adeno-associated virus (rAAV)-Cre-GFP was injected into *Fgf21^loxp^* mice via caudal vein one month before the MI surgery to inhibit FGF21 expression in mice, as the *Fgf21* KO mice. The validation of virus infection effect and KO model is shown in Figure S3. In our study, we performed *Fgf21* KO mice with AET intervention (KME group). The mortality of the mice in the KME group was up to 60% (Figure S4), especially during the adaptive training. Compared with the MI with AET group (MAE), we hardly detected the protein expression of FGF21 in the *Fgf21* KO mice (*p* < 0.01, Figure 2F). In addition, the CVF level (*p* < 0.01) and the protein expressions of COL-I and COL-III increased (both *p* < 0.01) significantly, in contrast, EF and FS decreased (both *p* < 0.01). These results showed that inhibition of FGF21 protein expression reduced the AET-induced improvement of cardiac function and fibrosis.

### 2.2. rhFGF21 and/or AICAR Inhibitedthe Activation of TGF-β1-Smad2/3-MMP2/9 Signaling Pathway, Promoted CFs Apoptosis and Antioxidant Capacity, Reduced Collagen Synthesis in CFs with H_2_O_2_-Treatment

**Figure 3:**
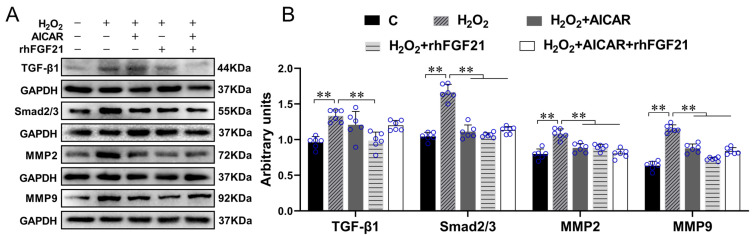
rhFGF21 and/or AICAR inhibited the activation of the TGF-β1-Smad2/3-MMP2/9 signaling pathway in CFs with H_2_O_2_ treatment. (**A**) The protein expressions of TGF-β1, Smad2/3, MMP2, and MMP9 in CFs. (**B**) Statistical analysis. Data are expressed as mean ± Standard Deviation (SD), *n* = 6. Statistical method: one-way analysis of variance (ANOVA). ** *p* < 0.01. rhFGF21, Recombinant human FGF21; AICAR, 5-Aminoimidazole-4-carboxamide ribonucleotide; CFs, cardiac fibroblasts; C, Untreated control group.

FGF21 has been reported to improve cardiac remodeling by inhibiting cardiac fibrosis [31,32]. However, the mechanism is still not well known. Fibrosis occurrence is closely related to the imbalance between proliferation and apoptosis of CFs and increased collagen secretion [33,34]. Therefore, we isolated and cultured the CFs from the neonatal mice hearts and explored the possible mechanism of FGF21 on inhibiting cardiac fibrosis. Oxidative stress is one of the causes of cardiac fibrosis in the injured heart [35]; we treated the CFs with H_2_O_2_ to mimic the ischemic myocardium and detected the effect of FGF21 on the activation of TGF-β1-Smad2/3 signaling in the CFs. Western blotting results showed that compared with control, H_2_O_2_ significantly increased the protein expression levels of TGF-β1, Smad2/3, MMP2 and MMP9 of CFs (all *p* < 0.01), which were inhibited by rhFGF21 intervention (all *p* < 0.01). AICAR, an activator of adenosine monophosphate (AMP)-activated protein kinase (AMPK), is often used to mimic the exercise effects in in vitro experiments. It has been demonstrated that AICAR could increase FGF21 production and release from cardiomyocytes [36]. In the present study, we found intervention of AICAR alone or combined with rhFGF21 reduced H_2_O_2_-induced increase of Smad2/3, MMP2 and MMP9 (all *p* < 0.01, Figure 3A,B). These results indicated both rhFGF21 and AICAR could significantly inhibit H_2_O_2_-induced activation of the TGF-β1-Smad2/3-MMP2/9 signaling pathway.

**Figure 4:**
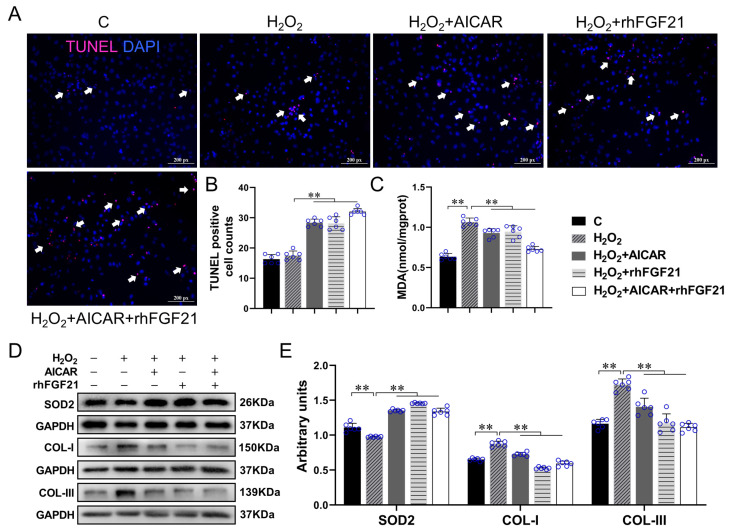
rhFGF21 and/or AICAR induced CFs apoptosis and reduced oxidative stress and fibrosis levels in CFs with H_2_O_2_ treatment. (**A**) TUNEL staining; the positive apoptotic particles showed red fluorescence, the nuclei showed blue, scale bar = 200 px; (**B**) statistical analysis of TUNEL staining; (**C**) MDA content of CFs; (**D**) the protein expressions of SOD2, COL- I and COL- III in CFs. (**E**) Statistical analysis. Data are expressed as mean ± Standard Deviation (SD), *n* = 6. Statistical method: one-way analysis of variance (ANOVA). ** *p* < 0.01. MDA, Malondialdehyde; C, Untreated control group; AICAR, 5-Aminoimidazole-4-carboxamide ribonucleotide; rhFGF21, Recombinant human FGF21.

To verify the effects of FGF21 and AICAR on cell apoptosis, antioxidant capacity, and collagen synthesis, we performed the TUNEL staining and detected the levels of Malondialdehyde (MDA), SOD2, COL-I and COL-III in CFs with H_2_O_2_ treatment. As shown in Figure 4, compared with the control cells, the level of MDA content and the protein expressions of COL-I and COL-III increased significantly (all *p* < 0.01), in contrast, SOD2 level decreased significantly after H_2_O_2_ treatment (*p* < 0.01). rhFGF21 and/or AICAR treatment increased the number of TUNEL positive particles (all *p* < 0.01) and SOD2 protein expression (all *p* < 0.01), and reduced the levels of MDA (all *p* < 0.01), COL-I (all *p* < 0.01) and COL-III (all *p* < 0.01) significantly in the H_2_O_2_-treated CFs. These results showed that AICAR and/or rhFGF21 intervention significantly promoted the CFs apoptosis and antioxidant capacity, and reduced collagen production in CFs with H_2_O_2_ treatment.

### 2.3. Exercise Training Inhibited the Activation of the TGF-β1-Smad2/3-MMP2/9 Signaling Pathway, Enhanced Antioxidant Capacity, and Reduced Cell Apoptosis via FGF21 in the Heart of Mice with MI

**Figure 5:**
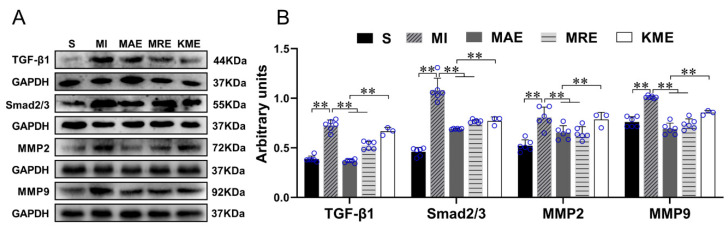
Exercise training inactivated the TGF-β1-Smad2/3-MMP2/9 signaling pathway via FGF21. (**A**) The protein expressions of TGF-β1, Smad2/3, MMP2 and MMP9. (**B**) Statistical analysis. Data are expressed as mean ± Standard Deviation (SD), *n* = 8 in S, MI, MAE and MRE groups and *n* = 4 in KME group. Statistical method: one-way analysis of variance (ANOVA). ** *p* < 0.01. S, Sham group; MI, MI-sedentary group; MAE, post-MI aerobic exercise training group; MRE, post-MI resistance exercise training group; and KME, the *Fgf21* KO mice with aerobic exercise training group.

Based on the results of in vitro experiment, we also evaluated the protein expression levels of TGF-β1, Smad2/3, MMP2 and MMP9 in vivo. As shown in Figure 5, the protein expression levels of TGF-β1, Smad2/3, MMP2, and MMP9 increased significantly in the MI group when compared with the S group (all *p* < 0.01). AET and RET down-regulated the protein expressions of TGF-β1 (both *p* < 0.01), Smad2/3 (both *p* < 0.01), MMP2 (both *p* < 0.01) and MMP9 (both *p* < 0.01) in the infarcted heart (all *p* < 0.01). In addition, compared with the MAE group, the protein expression levels of TGF-β1 (*p* < 0.01), Smad2/3 (*p* < 0.01), MMP2 (*p* < 0.01) and MMP9 (*p* < 0.01) increased in the KME group. These results indicated that exercise training partly inhibited the MI-induced activation of the TGF-β1-Smad2/3-MMP2/9 signaling pathway, and knockout of *Fgf21* attenuated the effects of AET.

**Figure 6:**
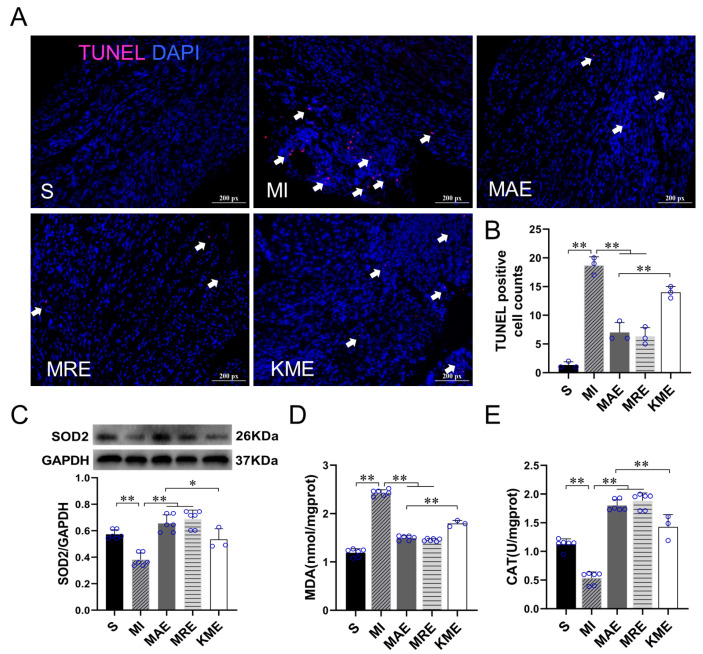
Exercise inhibited cell apoptosis and oxidative stress in the infarcted heart via FGF21. (**A**) TUNEL staining; the positive apoptotic particles showed red fluorescence, the nuclei showed blue, scale bar = 200 px; (**B**) statistical analysis of TUNEL staining; (**C**) the protein expression of SOD2; (**D**,**E**) MDA content and CAT activity. Data are expressed as mean ± Standard Deviation (SD), *n* = 8 in S, MI, MAE and MRE groups and *n* = 4 in KME group. Statistical method: one-way analysis of variance (ANOVA). * *p* < 0.05 and ** *p* < 0.01. S, Sham group; MI, MI-sedentary group; MAE, post-MI aerobic exercise training group; MRE, post-MI resistance exercise training group; and KME, the *Fgf21* KO mice with aerobic exercise training group.

We also detected the levels of oxidative stress and cell apoptosis in the mice with MI. The results showed that compared with the S group, the number of TUNEL positive cells (*p* < 0.01) and MDA content (*p* < 0.01) increased significantly, while protein expression of SOD2 (*p* < 0.01) and catalase (CAT) activity (*p* < 0.01) decreased in the MI group. Compared with the MI group, AET and RET reduced the number of TUNEL positive cells (all *p* < 0.01) and MDA content (all *p* < 0.01), and increased the expression of SOD2 protein (all *p* < 0.01) and CAT activity (all *p* < 0.01) in the infarcted heart. Moreover, compared with the MAE group, the number of TUNEL positive cells (*p* < 0.01) and MDA content (*p* < 0.01) increased, and SOD2 level (*p* < 0.05) and CAT activity (*p* < 0.01) decreased in the KME group (Figure 6A–E). These results pointed out that exercise can inhibit MI-induced oxidative stress and apoptosis, and knockout of *Fgf21* inhibited the protective effects of AET. We speculated that FGF21 would play a key role in exercise-induced cardioprotective effects following MI.

## 3. Discussion

**Figure 7:**
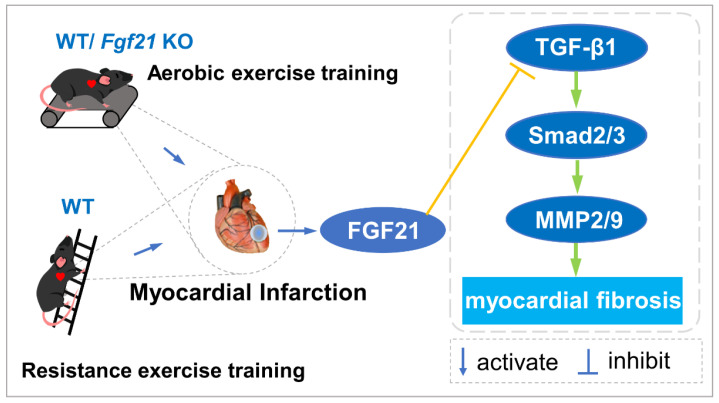
Aerobic and resistance exercise training up-regulated FGF21 inhibits the activation of TGF-β1-Smad2/3-MMP2/9 pathway and alleviates cardiac fibrosis following MI.

The salient findings of the present study include: (1) Both AET and RET alleviated cardiac dysfunction and fibrosis, and up-regulated FGF21 protein expression in the infarcted heart; (2) rhFGF21 and/or AICAR intervention inhibited the activation of the TGF-β1-Smad2/3-MMP2/9 signaling pathway, increased CFs apoptosis and reduced collagen production in CFs with H_2_O_2_ treatment; (3) Exercise training inactivated the TGF-β1-Smad2/3-MMP2/9 signaling pathway, alleviated cardiac fibrosis, enhanced antioxidant capacity and reduced cell apoptosis, and FGF21 plays an important role in the cardioprotective effect of exercise training (Figure 7).

Following MI, billions of cardiomyocytes are lost in response to ischemia. The adult mammalian heart is limited to repair after injury, and the lost cells are replaced by a fibrotic scar, which results in cardiac dysfunction. Exercise training has been proven to be an effective strategy of rehabilitation for MI patients [11,12]. Some studies detected the cardioprotective effects of different types of exercise models [6,7,9,10]. In this study, we confirmed that AET, RET, WBV, and ES enhanced the cardiac function of normal mice. In particular, the effects of AET and RET on cardiac function and cardiomyocytes physiological hypertrophy showed more significant than WBV and ES. According to this discovery, we further treated MI mice with AET and RET. In recent years, RET has been reported to have a beneficial effect on exercise tolerance in patients with hypertension and coronary artery disease. However, RET is not suitable for all patients, especially in patients with ventricular wall dyskinesia or severe ventricular arrhythmia. In the clinic, combined aerobic and resistance training or only aerobic training are often used for rehabilitation treatment [37,38,39]. Our results showed that both AET and RET could significantly improve cardiac function, which is consistent with previous research [6,7]. This result confirmed and suggested that both AET and RET could be included in the rehabilitation training of patients with MI, and patients can choose the suitable exercise mode according to their actual situation.

Cardiac injury triggers the activation and differentiation of CFs and disrupts the balance between cardiomyocytes and CFs [40]. Activated CFs increased extracellular matrix (ECM) deposition [41]. In the early stage of MI, ECM deposition exerts a protective effect for wound healing. However, excessive and continuous ECM deposition leads to cardiac fibrosis and dysfunction [42,43]. Some studies showed that the up-regulation of COL-I and COL-III following myocardial ischemia was related to cardiac fibrosis [4,44]. In our study, we confirmed MI increased CVF in the infarcted heart and up-regulated the protein expression levels of COL-I and COL-III. AET and RET reduced CVF as well as the levels of COL-I and COL-III. These results confirmed the anti-fibrosis effect of AET and RET.

FGF21 has been reported to be a cardioprotective factor [22,45]. In *Fgf21* knockout mice, myocardial fibrosis worsened after myocardial injury. [22,46]. FGF21 pre-treatment reduced Adriamycin-induced cardiac collagen precipitation [21]. Various exercise modes can up-regulate the protein expression of FGF21 [27,28,29]. Our results revealed that four types of exercise up-regulated FGF21 levels in the hearts of normal mice, and the protein expression level of FGF21 was closely related to cardiac function. In addition, AET and RET also up-regulated the FGF21 protein expression in the hearts of MI mice. To verify the importance of FGF21 in the exercise-induced improvement of cardiac function and fibrosis, we treated *Fgf21* KO mice with one kind of commonly used clinical rehabilitation method, the AET [39]. In this study, results showed knockout of *Fgf21* increased the protein expressions of COL-I and COL-III, and abrogated the AET-alleviated cardiac function and fibrosis in the infarcted hearts. Importantly, the mortality of infarcted *Fgf21* KO mice with AET intervention was higher than WT mice. It has been reported that FGF21 could prevent the generation of ROS and oxidative stress, relieving inflammation, alleviating cardiomyocyte apoptosis, autophagy, and cardiac fibrosis [21,22,23,24,25]. Exercise training could improve cardiac function and remodeling by preventing oxidative stress and cardiomyocyte apoptosis, and alleviating the development of cardiac fibrosis. Compared with the infarcted WT mice, under the same exercise intensity, knockout of FGF21 weakened the endogenous protective effects of FGF21 and exercise training, which resulted in excessive oxidative stress and cardiomyocyte apoptosis, increased the degree of fibrosis, decreased exercise tolerance and increased mortality. Therefore, it illustrated that FGF21 played an important role in mediating the protective effect of exercise training on the infarcted heart. Because of the insufficient number of *Fgf21* KO mice, we did not detect the role of FGF21 in all the types of exercise. According to the results, we speculated that FGF21 is necessary for the protective effects of exercise training in MI mice. We will further complete the work by carrying out RE in *Fgf21* KO MI mice in future work.

The TGF-β1-Smad2/3-MMPs signaling pathway plays a key role in the formation of cardiac fibrosis [4,17,47]. Over activation of TGF-β1 can promote the transformation of cardiac fibroblasts into myofibroblasts, and eventually leads to cardiomyocyte death, interstitial fibrosis, and increased degree of cardiac stiffness [14,48]. To better study the mechanism of FGF21 on inhibiting cardiac fibrosis, CFs were isolated from the hearts of neonatal mice and treated with H_2_O_2_, rhFGF21 and AICAR. It is well known that oxidative stress is the essential factor of myocardial injury and cardiac fibrosis after MI [40]. In our study, H_2_O_2_ treatment up-regulated the levels of TGF-β1, Smad2/3, MMP2, MMP9, and MDA, as well as COL-I and COL-III, and reduced SOD2 protein expression, suggesting the oxidative stress could activate TGF-β1-Smad2/3-MMPs signaling and increase the collagen secretion. It has been reported that AMPK agonist AICAR can promote TGF-β-induced apoptosis of pulmonary myofibroblasts and reduce collagen production [49]. Our results showed single intervention or combined intervention of AICAR and rhFGF21 improved oxidative stress by reducing MDA and increasing SOD2 level, inhibited the activation of TGF-β1-Smad2/3-MMPs signaling, and increased CFs apoptosis; meanwhile, it reduced the protein expression levels of COL-I and COL-III under the condition of H_2_O_2_ treatment. These results indicated both AICAR and FGF21 could reduce oxidative stress, promote CFs apoptosis, reduce collagen deposition under pathological conditions, and that the TGF-β1-Smad2/3-MMPs signaling would participate in this process. It was a pity for us not to detect the effects of AICAR or FGF21 intervention on CFs without H_2_O_2_ treatment. In the animal experiments, we verified exercise training could activate the TGF-β1-Smad2/3-MMPs signaling pathway and inhibit cardiac fibrosis through FGF21. It has been reported that TGF-β1 expression and MMP9 activity were significantly increased after MI in rats [50,51]. FGF21 can negatively regulate TGF-β1-induced Smad2/3 nuclear translocation, and reduce collagen precipitation and renal fibrosis [26]. In our study, we confirmed that AET and RET ameliorated MI-induced activation of the TGF-β1-Smad2/3-MMPs signaling pathway, which was attenuated by *Fgf21* knockout. Besides, it has been reported that FGF21 intervention could inhibit oxidative stress and apoptosis of damaged hearts [23,25]. To confirm this, in our study, we also found that MI increased cell apoptosis and reduced the antioxidant ability of the infarcted hearts, AET and RET reduced the cell apoptosis and increased the antioxidant ability, and knockout of *Fgf21* could weaken the beneficial effects of AET in MI mice.

The above results illustrated that FGF21 played an important role in the exercise-induced improvement of cardiac function and fibrosis. Exercise training increased FGF21 protein expression, inactivated the TGF-β1-Smad2/3-MMP2/9 signaling pathway, alleviated cardiac fibrosis, oxidative stress and cell apoptosis, and finally, improved cardiac function in mice with MI. In our future work, we will detect the effects of AET and RET on gene expression profiling and proteomics by using *Fgf21* knockout mice, and deeply reveal the role of FGF21 in the cardioprotective effect of exercise training.

## 4. Materials and Methods

### 4.1. Animals

Wildtype male C57BL/6J mice, three months old, were purchased from the laboratory animal center of the Xi’an Jiaotong University (Xi’an, China; No: SCXK (Shan) 2017-003). *Fgf21^loxP^* mice on the C57BL/6 background were purchased from The Jackson Laboratory (B6.129S6(SJL)-Fgf21tm1.2Djm/J, Bar Harbor, ME, USA). For inducing *Fgf21* KO mice, *Fgf21^loxP^* mice were injected with rAAV-CMV-EGFP-P2A-CRE-WPRE-bGHpA (rAAV-Cre, titer: 5.73 × 10^12^ vg/mL, 15 µL/20 g, BrainVTA Co., Ltd., Wuhan, China) via caudal vein four weeks before the establishment of the MI model. The genotype identification results are shown in Figure S1.

All mice were housed in the animal room of the institute of sports biology, Shaanxi Normal University. The temperature of the animal room was controlled at 25 °C and mice were given access to food and water freely under 12 h light/dark cycles. All mice were fed to 10 months old for experimental intervention. All surgical procedures and experimental protocols were performed with the Guide for Using Animal Subjects, and approved by the ethical committee of Shaanxi Normal University (approval number: 201916003; approved on 7 July 2019).

MI model was established by ligation of the left anterior descending (LAD) coronary artery. In brief, the 10-month-old mouse was anesthetized with isoflurane, fixed in the supine position and the chest was opened to expose the heart. LAD was ligated with 6.0 silk suture at the position approximately 2 mm under the junction of the left atrial appendage and pulmonary conus. Electrocardiogram (ECG) was used in the whole process to monitor the surgery, and ST-segment elevation was recognized as the sign of a successfully established model. On the seventh day after surgery, the echocardiography was performed, mice with similar infarct degrees were used in this study and divided into four groups: MI-sedentary group (MI, *n* = 8), MI with aerobic exercise training (AET) group (MAE, *n* = 8), MI with resistance exercise training (RET) group (MRE, *n* = 8) and FGF21 KO with AET group (KME, *n* = 4). Sham-operated mice without ligation were used as a control group (S, *n* = 8).

### 4.2. Exercise Protocols

After seven months feeding, WT mice were randomly divided into the control group (CON, *n* = 8), AET group (AET, *n* = 8), RET group (RET, *n* = 8), WBV group (WBV, *n* = 8), and ES group (ES, *n* = 8). Exercised mice were subjected to five weeks of different types of exercise training, including one-week adaptive training.

The AET protocol was performed on an animal treadmill (Zhenghua Technology Co., Hefei, China) as previously described [52]. On the first day, the training started at a speed of 8 m/min, for 10 min, and the speed increased to 12 m/min for 60 min on the fifth day. From the second week, the speed was kept at 12 m/min (oxygen consumption level is estimated at 76% of VO_2max_) for 60 min, five days per week for four weeks. Before and after each training, warm-up and cool down were carried out by running at a pace of 6 m/min for 5 min.

The RET program was modified from a published article [53,54]. Maximum carrying load was evaluated for each mouse by using a vertical ladder (1.0 m height, 1.0 cm intervals and 80° incline). Maximum carrying load was checked by ladder-climbing nine times with progressive loads. The heaviest load which mice could carry to climb successfully was viewed as the maximum carrying load. On the first day of the adaptive training, mice climbed the ladder with no load. The load increased with 15% maximum carrying load every day, and at the fifth day, up to 60% of the maximum load, climbing one time per set, nine sets per day with 1 min rest between sets. From the second week, the load was 75% of the maximum load, and this load was kept until the end of the training, three times per set, nine sets per day with 1 min rest between sets, for four weeks.

The WBV program was performed by using a vibration table as previously described and modified [55]: the frequency was 13 Hz, and the peak amplitude was 2.0 mm. In the first week, the time of vibration training was 10 min per day and up to 15 min per day from the second week to the end.

The skeletal muscle ES program was performed as described [56]: with anesthesia, the mice were fixed in a lying position. The needle was connected to the SDZ-II electronic acupuncture instrument, using continuous pulses with an electrical frequency of 20 Hz and a current of 1 mA. In the first week, the electrical stimulation program was performed 10 min per day, and from the second week, up to 15 min per day for four weeks.

According to the effects of different types of training on WT mice, MI mice were subjected to five weeks of AET or RET. The programs were performed as previous results [53,54,57].

AET program for MI mice: The first day of adaptive training was 10 m/min for 10 min, and increased to 10 m/min for 50 min on the fifth day. From the second week, the training started at 10 m/min for 60 min per day, for four weeks.

RET program for MI mice: the adaptive training is the same as normal mice. From the second week, the load was 75% of the maximum load and the load was kept until the training ended, climbing one time per set, nine sets per day for four weeks.

### 4.3. Cardiac Fibroblast Isolation and Cell Culture

Primary CFs were isolated from neonatal mice 1–3 days post-birth (dpb) according to the literature [58,59]. After being disinfected with alcohol, the heart was taken out from mice and the ventricular tissue was cut into small pieces and digested with digestion buffer (0.04% trypsin, 20 mM 2,3-Butanedione 2-monoxime (BDM), 0.08% Type II collagenase, phosphate-buffered saline (PBS)) at 37 °C. The cell suspension was collected and added an equal volume of complete medium to terminate digestion. After centrifugation at 1200 rpm/min, we discarded the supernatant and resuspended the cell precipitate in complete medium, and cultured the cells in the incubator (Thermo Model 371, Marietta, OH, USA) for 90 min. Cardiomyocytes and CFs were collected by differential centrifugation. We collected the CFs, and cultured them with 10% FBS-DMEM. The morphology of myocardial fibroblasts should be fusiform, triangular and polygonal.

We collected cells and inoculated them into 6-well plates. When cell density reached to 70–80%, CFs were treated with H_2_O_2_ (100 μM, 6 h, to establish a model of oxidative stress-induced injury) [60], 5-aminoimidazole-4-carboxamide-1-β-d-ribofuranoside (AICAR, Selleck Chemicals, Houston, TX, USA, 1 mM, 15 h, to mimic the exercise effect) [49], and FGF21 Recombinant protein (rhFGF21, Selleck Chemicals, Houston, TX, USA, 100 ng/mL, 15 h) [61].

### 4.4. Echocardiographic Measurement

Before and after the whole exercise process, echocardiography was detected using an ultrasound cardio tachograph (VINNO 6 VET, VINNO, Suzhou, China) to assess cardiac functions. The mouse was fixed in the supine position and inhalation anesthetized with isoflurane mixed with oxygen (1:5). LVIDs, LVIDd and EF were recorded. The FS was calculated as (LVIDd−LVIDs)/LVIDd × 100%. Then, mice were sacrificed, the heart was quickly collected, and fixed in cold 4% formaldehyde or liquid nitrogen for subsequent experiments.

### 4.5. Histological Staining and Analysis

The heart tissue, fixed with paraformaldehyde for 48 h, was used for histological staining. After being washed with water, heart samples went through dehydration, transparent and paraffin embedding, then were cut into 5 μm thick microtome sections. Masson staining and Sirius red staining were performed on myocardial tissue sections to evaluate the degree of myocardial fibrosis. The collagen volume fraction was analyzed by Image Pro Plus analysis software (IPWIN Media Cybernetics, Inc., Rockville, MD, USA), and the percentage of CVF was calculated as collagen area/total area of myocardial tissue × 100%.

### 4.6. Immunofluorescence Staining

Heart tissue fixed with 4% paraformaldehyde was also used to perform frozen section (10 μm). After being fixed in cold acetone for 5–10 min, the frozen sections were washed with PBS (pH = 7.2), then incubated with 5% bovine serum albumin (BSA) for 1 h. Then, sections were incubated with rabbit polyclonal antibody Laminin (1:1000, Abcam, Waltham, MA, USA) at 4 °C overnight. On the next day, after washing with PBS, the sections were incubated with the tetramethyl rhodamine isothiocyanate (TRITC)-labeled goat anti-rabbit antibody (1:100, Jackson Immunoresearch, West Grove, PA, USA) at room temperature in darkness for 1.5 h. Then, they were washed with PBS and sealed with an anti-fluorescence quench agent. The images were observed and collected by using a fluorescence microscope (Nikon Eclipse 55i, Tokyo, Japan). Three sections of each sample were scanned, and 20 fields per section were viewed under a microscope. Image J software (National Institutes of Health, Baltimore, MD, USA) was used to analyze the cross-sectional area (CSA) of cardiomyocytes.

### 4.7. TUNEL Staining

Cell apoptosis in heart tissues and CFs was detected by using a terminal deoxynucleotidyl transferase (TdT) dUTP nick-end labeling (TUNEL) assay (Beyotime, Shanghai, China). Sections of paraffin embedded tissue were dewaxed to water, incubated with protease K at 37 °C for 30 min, and washed with PBS. Then, sections were incubated with prepared TUNEL solution (TdT enzyme: fluorescent labeling solution = 1:9) at 37 °C for 60 min. The nuclei were stained with 4′,6-Diamidino-2-phenylindole (DAPI, 1:800, US EVERBRIGHT, Suzhou, China). After being washed with PBS, sections were sealed, and the images were captured using the fluorescence microscope (Nikon Eclipse 55i, Tokyo, Japan).

For CFs, the cell climbing sheets were fixed with 4% paraformaldehyde for 30 min, then incubated with 0.3% Triton X-100 solution for 5 min, and washed with PBS. The follow-up operation is the same as paraffin section.

### 4.8. Kit Assays

MDA content and CAT activity in myocardial tissue homogenate and CFs cells were detected by using the assay kits (Jiancheng Biotech, Nanjing, China) according to the manufacturer’s protocols.

### 4.9. Western Blotting

The heart samples and CFs harvested were lysed in the lysate mixture (RIPA: PMSF: phosphatase inhibitor = 100:1:1) and crushed by tissue homogenizer (FLUKO, Shanghai, China) and ultrasonic cell disruptor (Jining Tianhua Ultrasonic Electronic instrument Co., Jining, China). The supernatant was extracted and followed by protein quantification and denaturation. The total protein was separated by SDS-PAGE and then transferred to nitrocellulose membranes (Millipore, Bredford, MA, USA). The membranes were incubated in 3% BSA at room temperature for 1.5 h and then incubated with primary antibodies: FGF21 (1:1000 dilution, Abcam, Waltham, MA, USA), TGF-β1 (1:1000, Proteintech, Rosemont, IL, USA), Smad2/3 (1:1000, Abcam, Waltham, MA, USA), MMP2 (1:1000, Proteintech, Rosemont, IL, USA), MMP9 (1:1000, Proteintech, Rosemont, IL, USA), COL-I (1:500, Affinity Biosciences, Cincinnati, OH, USA), COL-III (1:500, Proteintech, Rosemont, IL, USA), SOD2 (1:1000, GeneTex, Irvine, CA, USA). GAPDH was used as a loading control for protein normalization. The next day, the membranes were washed with Tris-Buffered Saline and Tween 20 (TBST) three times, and then incubated with the horseradish-peroxidase (HRP)-conjugated secondary antibody at room temperature for 1 h. Reactive bands were detected using enhanced chemiluminescence reagent (Bio-Rad, Berkeley, CA, USA), analyzed using a digitalized Bio-Rad ChemiDocTM MP Imaging system (Universal Hood III, Bio-Rad, Berkeley, CA, USA), and quantified using Imagelab software 5.1 (Bio-Rad, Berkeley, CA, USA).

### 4.10. Statistical Analysis

Image Lab Software 5.1 (Bio-Rad, CA, USA) was used to analyze the Western blotting results, GraphPad Prism 8.0.2 (GraphPad Software, La Jolla, CA, USA) was used for drawing the graphs. SPSS 21.0 statistical software (IBM Company, Armonk, NY, USA) was used for data analysis, and the differences between groups were determined by one-way analysis of variance (ANOVA). Values were expressed as mean ± Standard Deviation ($\bar{X}$ ± SD) and significance levels were set at *p* < 0.05 and *p* < 0.01.

## Data Availability

The data presented in this study are available in the article and Supplementary Materials.

## Supplementary Materials

The following are available online at https://www.mdpi.com/article/10.3390/ijms222212341/s1.
