# Supplementary material for: Exercise Training Alleviates Cardiac Fibrosis through Increasing Fibroblast Growth Factor 21 and Regulating TGF-β1-Smad2/3-MMP2/9 Signaling in Mice with Myocardial Infarction
Source: Int J Mol Sci. 2021 Nov 15;22(22):12341. doi: 10.3390/ijms222212341 (PMC8623999; doi:10.3390/ijms222212341)
Supplement: Supplementary file 1 [file ijms-22-12341-s001.zip › ijms-1381822-supplementary.pdf]

1 Effects of different types of exercise training on cardiomyocyte size, heart weight and cardiac function in normal mice

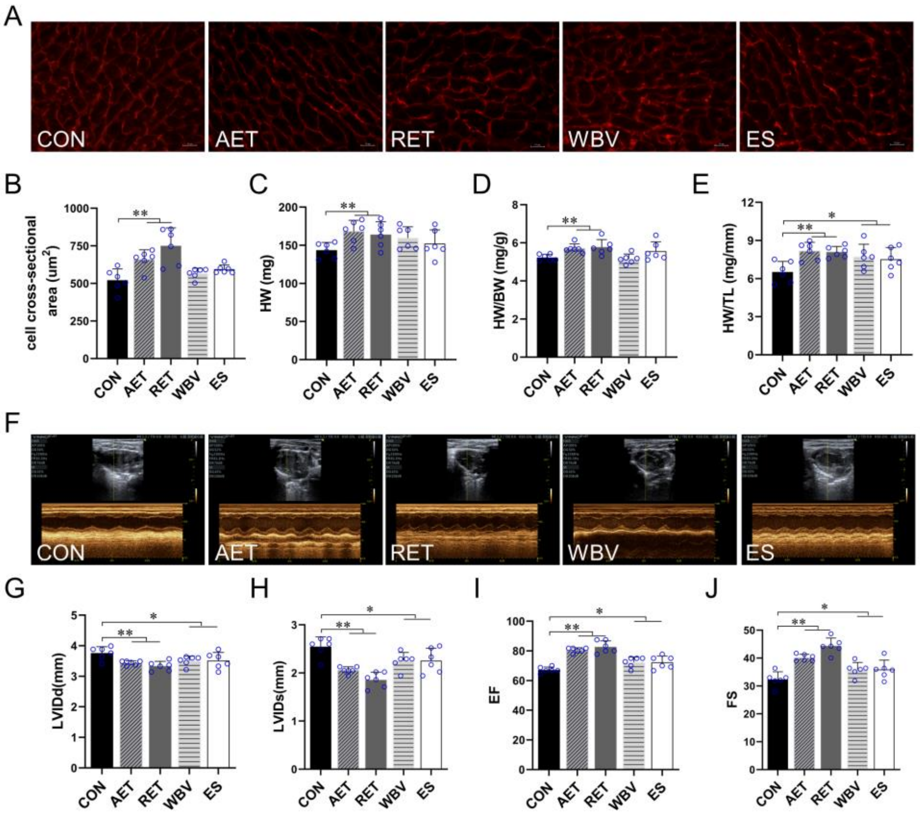

Figure. S1 Different types of exercise training increased cardiomyocyte size and heart weight, improved cardiac function in normal mice.

(A-B) Laminin staining was performed to label cell membrane of cardiomyocytes in the left ventricular of normal mice. (C-E) Heart weight, ratios of heart weight to bodyweight or tibia length in mice. (F-J) Statistical analysis of echocardiography results and cardiac function indexes in mice. Data are expressed as mean ± Standard Deviation (SD), n = 8. Symbols indicate * $P<0.05$ and ** $P<0.01$. HW, heart weight; HW/BW, the ratio of heart weight / body weight; HW/TL, the ratio of heart weight / tibial length; LVIDd, left ventricular internal diameter at end diastole; LVIDs, left ventricular internal diameter at end systole; EF, left ventricular ejection fraction and FS, left ventricular fractional shortening. Abbreviations of groups: CON, control group; AET, aerobic exercise training group; RET, resistance exercise training group; WBV, whole body vibration exercise group and ES, skeletal muscle electrical stimulation group.

2 Effects of different types of exercise training on FGF21 protein expression in mice

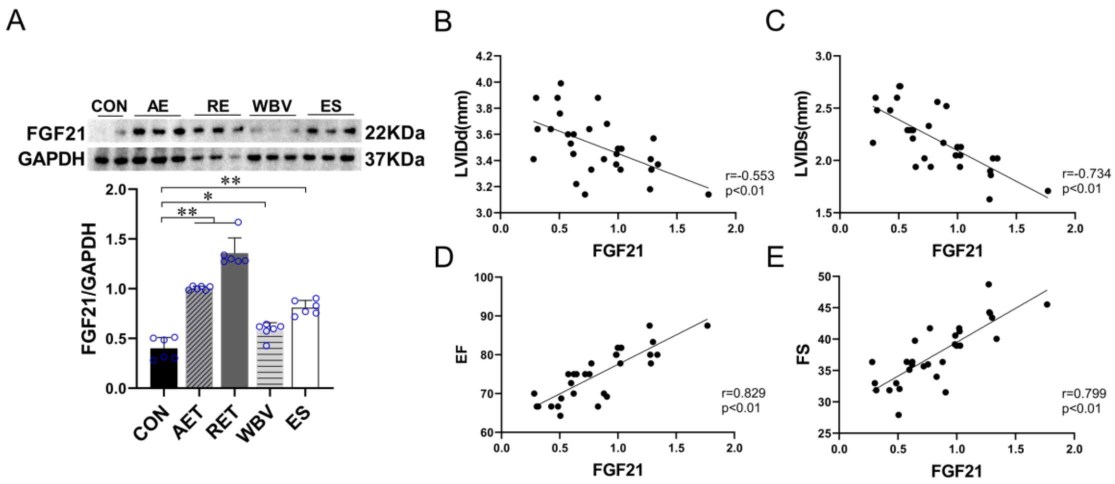

Figure.S2 Correlation analysis between cardiac FGF21 protein expression and cardiac function after different exercise. (A) Expression of FGF21 protein in mouse myocardium. (B-E) Correlation analysis between myocardial FGF21 protein expression and cardiac function. Data are expressed as mean ± Standard Deviation (SD), n = 8. Symbols indicate * *P*<0.05 and ** *P*<0.01. LVIDd, left ventricular internal diameter at end diastole; LVIDs, left ventricular internal diameter at end systole; EF, left ventricular ejection fraction and FS, left ventricular fractional shortening. Abbreviations of groups: CON, control group; AET, aerobic exercise training group; RET, resistance exercise training group; WBV, whole body vibration exercise group and ES, skeletal muscle electrical stimulation group.

3 Identification results of FGF21 knockout mice

Table S1 Mouse tail identification primer sequence

Gene	Primer	Sequence
FGF21	Forward	5’- GAAACAAAGCTTCAAAATAGGG-3’
	Reverse	5’- AGTAGGGGTCAGACGTGGTG-3’

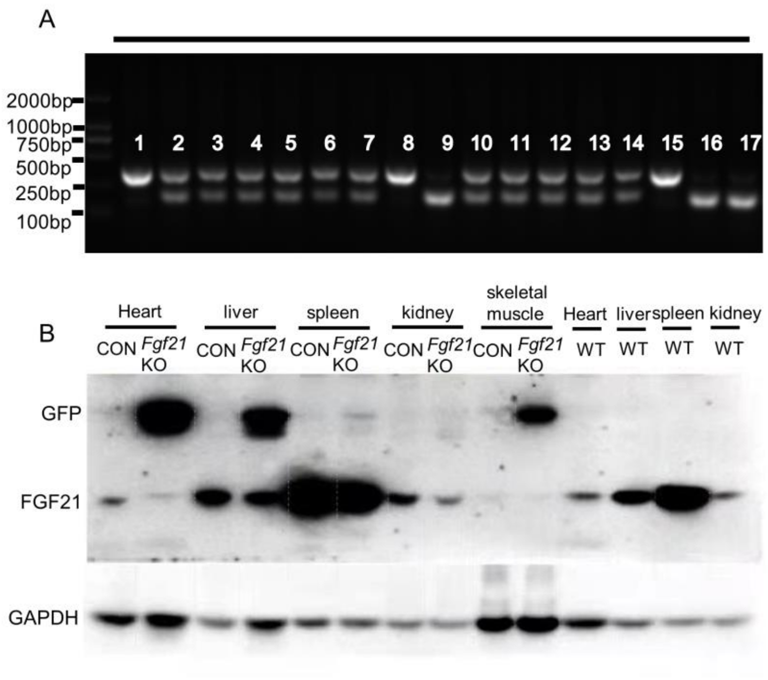

Figure S3. Identification results of FGF21 knockout mice.

(A) The electropherogram of mouse tail DNA. The mouse tail was lysed for extracting DNA. After PCR, the amplificated products were used for horizontal electrophoresis to identify genotypes.Only a band at 352bp is a mutant (loxp$^{+/+}$), a band at 352bp and a band at 179bp is heterozygous (loxp$^{+/-}$), and a band at 179bp is a wild type (loxp$^{-/-}$). (B) The expression levels of GFP and FGF21 in different organs after injection of rAAV-Cre-GFP virus. CON, virus empty body group; *Fgf21 KO*, rAAV-Cre-GFP virus injection group; WT, wild type control group.

4 Survival rate

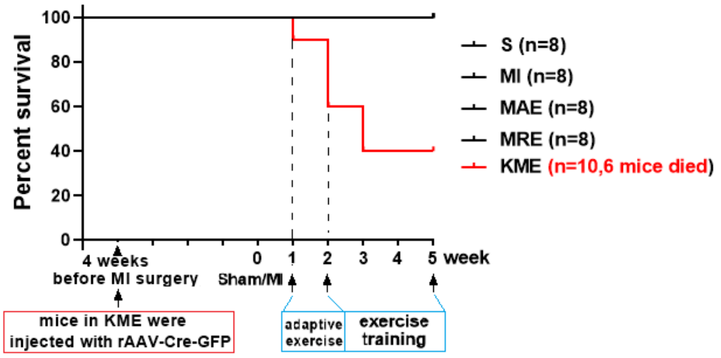

Figure S4. Survival rate.

S, Sham group; MI, MI-sedentary group; MAE, post-MI aerobic exercise training group; MRE, post-MI resistance exercise training group; and KME, the FGF21 KO with aerobic exercise training group.
